# Lack of Association Found between* Helicobacter pylori* Infection and Diarrhea-Predominant Irritable Bowel Syndrome: A Multicenter Retrospective Study

**DOI:** 10.1155/2016/3059201

**Published:** 2016-07-17

**Authors:** Feng Xiong, Man Xiong, Zonghui Ma, Senxiong Huang, Aimin Li, Side Liu

**Affiliations:** Guangdong Provincial Key Laboratory of Gastroenterology, Department of Gastroenterology, Nanfang Hospital, Southern Medical University, Guangzhou 510000, China

## Abstract

*Aims*. The association between* Helicobacter pylori* (*H. pylori*) infection and diarrhea-predominant irritable bowel syndrome (IBS-D) is still controversial. Here we performed a retrospective study to explore this issue.* Methods*. A total of 502 inpatients with Rome III confirmed IBS-D and known* H. pylori* status from 8 hospitals were enrolled.* H. pylori-*positive patients, hospitalized in the recent year, were followed up to evaluate the effects of* H. pylori *eradication on IBS-D clinical course.* Results*. Of the 502 IBS-D patients, 206 were* H. pylori*-positive, with an infection rate that has no significant difference with that of the general population in Guangdong province (*p* = 0.348). For patients followed up, no significant differences were noted as to overall symptoms (*p* = 0.562), abdominal pain/discomfort (*p* = 0.777), bloating (*p* = 0.736), stool frequency (*p* = 0.835), or stool characteristics (*p* = 0.928) between the* H. pylori-*eradicated group and the control group. The results were the same in long-term follow-up patients except the improvement of bloating, which showed that the bloating score in the* H. pylori-*eradicated group was significantly lower (*p* = 0.047).* Conclusions*. No significant correlation between* H. pylori* infection and IBS-D was noted. Overall, IBS-D patients may not benefit from* H. pylori* eradication.

## 1. Introduction

Irritable bowel syndrome (IBS) is a common gastrointestinal disorder. The disease typically presents as abdominal pain/discomfort, accompanied by changes in bowel habits and with features of disordered defecation. Concomitantly, there is no evidence for any structural or organic lesions in the gastrointestinal tract. The Rome III criteria are now generally accepted as confirming IBS [1]. IBS affects up to 20% of the population in western countries [2]. In China the reported prevalence is much lower, about 4–6% [3, 4]. As there is a paucity of effective treatments for IBS, the disease has substantial influence on patients' quality of life [5, 6] and socioeconomic status [7]. Thus, novel therapies in IBS are urgently needed.

The quest for better care for IBS patients is handicapped by a lack of insight into the underlying pathogenic mechanisms. Previous studies suggested that abnormal brain-gut interactions, alteration of intestinal flora, chronic low-grade mucosal inflammation, and psychological disturbance may be involved in the pathophysiology of IBS [8–11]. These processes provoke altered bowel motility and increase mucosal permeability and visceral hypersensitivity, which then give rise to the clinical symptoms on IBS. Increased knowledge of the mechanisms involved may guide development of a rational therapy. It has been proposed that changing the composition of the intestinal flora may become important in this respect.

As a bacteria specialized colonizing on the gastric mucosa,* H. pylori* is known as the main cause of chronic gastritis, peptic ulcer, gastric carcinoma, and gastric mucosa-associated lymphoid tissues lymphoma [12–14]. Furthermore,* H. pylori* may have a role in extragastric disorders [15, 16], probably by triggering systemic inflammatory responses. However, the association between* H. pylori* infection and IBS is controversial [4, 17–19]. Considering that gastrointestinal infection is a main reason of IBS-D, some studies [19, 20] suggested that* H. pylori* infection may play a role especially in IBS-D, but the relevant clinical study is still lacking.

The abovementioned considerations prompted us to perform a retrospective investigation into the association between* H. pylori* status and IBS-D.

## 2. Materials and Methods

### 2.1. Patients

We retrieved the medical records of inpatients that were diagnosed with IBS in 8 hospitals of the Guangdong province in China, from January 2012 to July 2015. Subsequently, those patients who met the Rome III criteria for IBS-D were identified. Patients with the following conditions were excluded: (1) being older than 70 years or younger than 18 years at the time of diagnosis; (2) having IBS-D associated with peptic ulcer, gastrointestinal tumor, abdominal surgery, a psychological disease, or hyperthyroidism; (3) those who had not undergone* H. pylori* testing. General information including name, gender, age, and body mass index (BMI) was recorded, as was the date of admission to the hospital and patients' contact information. Also retrieved were the results of* H. pylori* test, as well as the abdominal pain/discomfort score, bloating score, stool frequency, and stool characteristic before eradication.

### 2.2. Follow-Up and Symptom Assessment

**Figure 1:**
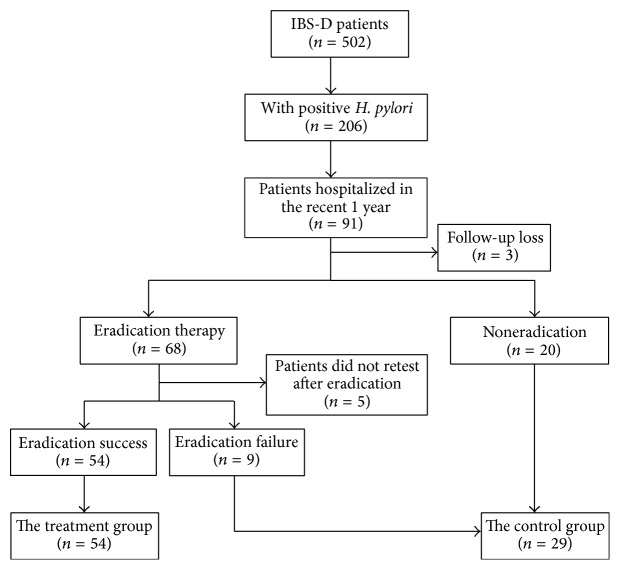
Flow chart of the analyses performed in this study. IBS-D: diarrhea-predominant irritable bowel syndrome;* H. pylori*:* Helicobacter pylori*.

To reduce memory bias, only the* H. pylori*-positive IBS-D patients who were hospitalized between August 2014 and July 2015 were followed up. All of the patients underwent* H. pylori* eradication using the quadruple therapy (clarithromycin, 500 mg, 2/d  + amoxicillin and clavulanate potassium, 0.457 g, 2/d  + esomeprazole, 20 mg, 2/d  + bismuth potassium citrate, 0.6 g, 2/d) for 2 weeks. The review results of the* H. pylori* as well as the abdominal pain/discomfort score, bloating score, stool frequency, and stool character were recorded after eradication For the study analysis, patients with* H. pylori* eradicated successfully were assigned to the* H. pylori*-eradicated group. Patients for whom* H. pylori* eradication failed, or who did not undergo attempted* H. pylori* eradication, were assigned to the control group. Comparisons were conducted between the two groups to assess the efficacy after* H. pylori* eradication (Figure 1).

The efficacy of overall symptoms was categorized as complete, partial, or nonresponse. A complete response was considered the complete normalization of all of the IBS-D symptoms. A partial response was defined as a significant improvement in abdominal pain/discomfort and bloating, with the frequency of stool reducing to less than half before intervention and with the score of the stool decreasing by more than 1 point according to the Bristol stool scale. Patients were recorded as nonresponders if the improvement of the overall symptoms did not reach the standard of partial response. The patients defined as final responders comprised those with a complete response or a partial response. The efficacy of* H. pylori* eradication on IBS-D was calculated by dividing the number of responders by the total number of patients in each group. The improvement of each symptom was analyzed to assess the efficacy further. The rating standard of abdominal pain/discomfort and bloating is as follows: asymptomatic, 0 points; mild symptoms not affecting daily life, 1 point; moderate symptoms affecting daily life but not significantly, 2 points; severe symptoms seriously affecting the normal life, 3 points [21]. Diarrhea rating is based on the number of stool frequencies in patients per day, and we take the maximum daily stool frequency as the records. Stool character rating: it referred to the Bristol stool scare to scoring; the scores were recorded when the patient's stool corresponded to the subtype in the scan.

### 2.3. Statistical Analysis

All statistical analyses were performed using SPSS software (version 20.0; SPSS, Chicago, IL, USA). Measurement data were expressed as mean ± standard deviation (mean ± SD) representation; count data were displayed as rate; A binomial test was used to evaluate the difference between the sample rate and the population rate of* H. pylori* infection. Measurement date between the* H. pylori-*eradicated group and the control group were analyzed using a *t*-test. However, count data were analyzed using a chi-squared test. *p* values < 0.05 were considered statistically significant.

## 3. Results

### 3.1. Infection Rate of* H. pylori* in IBS-D Patients

The records of 502 patients (237 men, 265 women) with IBS-D, coming from 8 different hospitals, were used for the present study. Of these, 206 were* H. pylori*-positive, with an infection rate of 41.04%, which is even a little lower than that of the general population in Guangdong province (42.01%) [22], and the difference is not statistically significant (*p* = 0.348).

**Figure 2:**
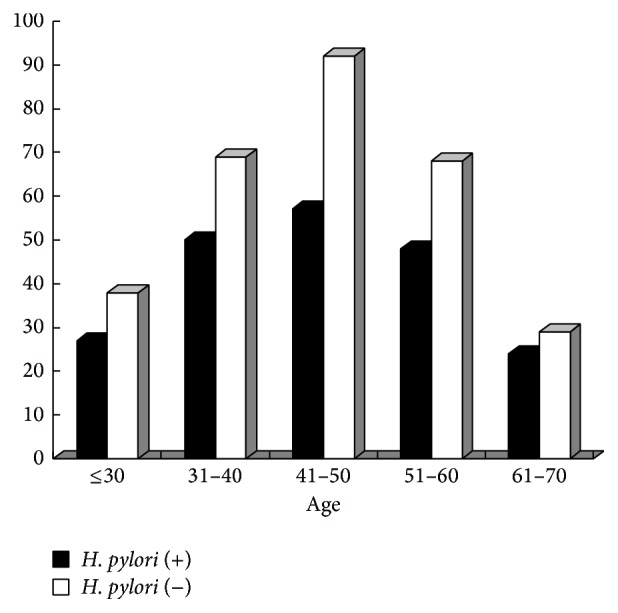
Age-related prevalence of IBS-D in cases with and without* H. pylori* infection.

Furthermore, we stratified the IBS-D patients according to the age. For the age categories, ≤30, 31–40, 41–50, 51–60, and 61–70,* H. pylori* infection rates were 41.54% (27/65), 42.02% (50/119), 38.26% (57/149), 41.38% (48/116), and 45.28% (24/53), respectively (Figure 2). The results showed that patients between 41 and 50 years of age had the highest prevalence of IBS-D, but they had the lowest infection rate of* H. pylori*, although no significant difference about the infection rate was found among these categories (*p* = 0.920). These results suggest that* H. pylori* status may not be important with respect to IBS-D.

### 3.2. The Development of Syndromes after* H. pylori* Eradication

There were 91* H. pylori*-positive patients hospitalized from August 2014 to July 2015. Only 3 patients were lost to follow-up, and 20 patients did not undergo* H. pylori* eradication. In the patients that underwent eradication, 5 did not retest after eradication and thus were excluded from further analysis, 9 patients experienced eradication failure, and for 54 patients, attempted eradication was a success. Thus, finally there were 54 patients in the* H. pylori*-eradicated group and 29 patients in the control group. There were no statistical differences in gender ratios, age, BMI, follow-up time and abdominal pain/discomfort score, bloating score, stool frequency, and stool character between these two groups (Table 1).

The number of responders in the* H. pylori-*eradicated group was 30 (11 with a complete response and 19 with a partial response), with an effective rate of 55.56%, while in the control group there were 14 responders (4 with a complete response and 10 with a partial response), with an apparent effective rate of 48.28%. There was no significant difference in the rate of response with respect to successful IBS-D treatment between the* H. pylori-*eradicated group and the control group (*p* = 0.526). We analyzed the improvement of the main IBS-D symptoms further and found that there was no significant difference on abdominal pain/discomfort score, bloating score, stool frequency, and stool characteristic, too (Table 2).

### 3.3. The Development of Syndromes after* H. pylori* Eradication of Long-Term Follow-Up

Previous study suggested that the efficacy of* H. pylori* eradication may not show immediately and a long-term follow-up should be taken to determine the efficacy after* H. pylori* eradication [23]. Thus, we also compared the results among patients with follow-up time longer than 3 months. Finally 42 and 22 patients were assigned to the* H. pylori*-eradicated group and the control group, respectively. Between these groups, there were no statistical differences in gender ratios, age, BMI, follow-up time and abdominal pain/discomfort score, bloating score, stool frequency, and stool characteristic (Table 3).

The number of responders in the* H. pylori*-eradicated group was 25 (8 with a complete response and 17 with a partial response), receiving an apparent effective rate of 59.52%, while, in the controlgroup, the number of responders was 11 (3 with a complete response and 8 with a partial response), receiving an efficacy of 50.00%. No statistical difference was found between the two groups (*p* = 0.526). Also, we did not find any significant differences on abdominal pain/discomfort score, stool frequency, and stool characteristic after* H. pylori* eradication, but the difference of bloating score between the two groups is significant, and the improvement of bloating in the* H. pylori*-eradicated group was obviously better than that of the control group (*p* = 0.047) (Table 4).

Based on the results above, we concluded that IBS-D patients seem not to benefit from* H. pylori *eradication, with the exception of the effect on bloating seen in long-term follow-up. Considering that bloating is not a primary efficacy endpoint, our data fail to demonstrate a relevant effect of* H. pylori *eradication on the clinical course of IBS-D.

## 4. Discussion

IBS is a substantial medical challenge to society, and the development of a novel treatment for this disease is frustrated by the lack of insight into its etiology and pathogenesis. In the present study we explored the potential association between* H. pylori* and IBS-D. Previous basic studies have suggested that the systemic inflammation provoked by CagA (cytotoxin-associated gene A) and VacA (vacuolating cytotoxin) of* H. pylori* may link this bacterium to the pathogenesis of IBS [19]. The proposed mechanism has 3 main features, as follows. Firstly, systemic inflammation stimulates mast cells, T lymphocytes, and enterochromaffin cells, which would secrete proinflammatory neurotransmitters like 5-HT, substance P (SP), and calcitonin gene-related peptide (CGRP), and these in turn influence the brain-gut axis [24, 25]. Secondly, the inflammation evoked by* H. pylori* can act directly on the intestinal mucosa, increasing intestinal permeability, and can cause alterations in the gut flora [19, 26]. Thirdly,* H. pylori*-induced inflammation then augments stress response in patients and influences the hypothalamus-pituitary-adrenal (HPA) axis [25, 27]. The mechanisms above would cause visceral hypersensitivity and increased bowel motility, resulting in patients with abdominal pain/discomfort, bloating, and diarrhea. Herein, we attempted to test this hypothesis through a retrospective analysis of the effects of* H. pylori* eradication in a large patient population from 8 hospitals in the Guangdong province of China. The results, however, do not support a role of* H. pylori* in the pathogenesis of IBS-D.

We observed that the infection rate of* H. pylori* in IBS-D patients was below 50%, meaning that the percentage of* H. pylori*-positive patients was less than that of the* H. pylori*-negative patients. Furthermore, the overall infection rate of* H. pylori* in IBS-D patients in the present study has no significant difference from that of the general population in the Guangdong province. Finally, as shown in the Figure 1 we found that patients between 41 and 50 years of age had the highest prevalence of IBS-D, but they had the lowest infection rate of* H. pylori*, though there is no difference among these groups. So, we considered that* H. pylori *may not play a key role in IBS-D. This conclusion is in agreement with the reports from Shanghai of China and Japan [4, 18] but contradicts a study from Taiwan [17] that reported that, in IBS patients, the presence of dyspepsia is associated with* H. pylori* infection. What cannot be ignored, however, is that in the latter study the subjects were IBS patients complicated by functional dyspepsia (FD), while FD now is proven to be closely correlated with* H. pylori* infection.

In the present study,* H. pylori* eradication did not improve the overall symptoms or abdominal pain/discomfort, stool frequency, and stool characteristic in IBS-D patients. As shown in Tables 3 and 4, although the efficacy rate is higher in the* H. pylori*-eradicated group, there was no significant difference between the two groups in overall follow-up, nor in long-term follow-up. This suggests that there is no place for* H. pylori* eradication in the clinical management of main symptoms of this disease. Alekseenko et al. [28] reported differently, demonstrating that 63.5% of IBS patients showed clinical improvement after* H. pylori* eradication. Strikingly, in that study the subjects also were patients suffering from IBS associated with FD and thus cannot be directly compared to the study we present here. Furthermore this study was limited to uncontrolled monitoring of symptoms, not involving a control group, different from the study presented here.

We did find in long-term follow-up time a benefit from* H. pylori* eradication with respect to bloating. This effect may derive from the antibiotic therapy mediating* H. pylori* eradication, which may decrease the number of bacteria that can produce methane and hydrogen in the intestine thus relieving bloating symptoms [29]. In addition, successful eradication can prevent* H. pylori* from producing metabolites that can stimulate the nervous reflex and hormone secretion in the stomach, resulting in normalization of gastric motility. Such an effect may be relevant in this context as normalization of gastric motility has been described to effectively counteract bloating [30].

One of the limitations is the limited sample size. Although the total number of IBS-D patients involved was large, those who were followed up were relatively few. The small sample size may be the reason that the* H. pylori*-eradicated group had a higher efficacy rate but did not reach statistical significance. Then, although the patients followed up were hospitalized during the recent year, we still could not avoid a memory bias that could affect the evaluation of the efficacy and the final results.

In conclusion, the results of the present study indicate that* H. pylori* infection may not play an important role in IBS-D, and IBS-D patients seem to not benefit from* H. pylori *eradication except bloating in patients of long-term follow-up. Larger prospective studies assessing the efficacy in IBS-D patients after* H. pylori* eradication are required.

## Figures and Tables

**Table 1:** Baseline characteristics in the *H. pylori*-eradicated group and the control group in patients of follow-up.

	*H. pylori*-eradicated group	Control group	*p* value
Sex (male/female), *n*	26/28	16/13	0.556
Age (yr)	44.49 ± 12.30	46.69 ± 13.49	0.911
BMI (kg/m^2^)	21.64 ± 2.64	21.31 ± 2.24	0.915
Follow-up time (months)	7.00 ± 3.60	7.10 ± 3.53	0.900
Abdominal pain score	1.76 ± 0.70	1.72 ± 0.65	0.824
Bloating score	1.52 ± 0.59	1.42 ± 0.51	0.607
Stools per day	5.07 ± 1.13	5.07 ± 0.92	0.983
Stool character score	6.17 ± 0.72	6.06 ± 0.65	0.544

Data are presented as mean ± SD or number.

*H. pylori*:  *Helicobacter pylori*; BMI: body mass index.

**Table 2:** Main study outcomes in the *H. pylori*-eradicated group and the control group in patients of follow-up.

	*H. pylori*-eradicated group	Control group	*p* value
Complete response	11/54	4/29	
Partial response	19/54	10/29	
Responders^*∗*^	30/54	14/29	0.562
Abdominal pain score	1.15 ± 0.92	1.21 ± 0.66	0.777
Bloating score	0.91 ± 0.67	1.00 ± 0.74	0.736
Stools per day	3.20 ± 1.50	3.28 ± 1.51	0.835
Stool character score	4.89 ± 1.31	4.86 ± 1.25	0.928

Data are presented as mean ± SD or number.

^*∗*^Responders included patients who showed complete or partial response.

*H. pylori*:  *Helicobacter pylori*.

**Table 3:** Baseline characteristics in the *H. pylori*-eradicated group and the control group in patients of long-term follow-up.

	*H. pylori*-eradicated group	Control group	*p* value
Sex (male/female), *n*	19/23	10/12	0.974
Age (yr)	45.88 ± 12.52	46.14 ± 10.91	0.247
BMI (kg/m^2^)	21.20 ± 2.84	21.61 ± 2.95	0.836
Follow-up time (months)	8.14 ± 3.16	8.41 ± 2.87	0.742
Abdominal pain score	1.74 ± 0.70	1.77 ± 0.69	0.851
Bloating score	1.44 ± 0.51	1.50 ± 0.53	0.803
Stools per day	5.07 ± 1.20	4.91 ± 0.97	0.586
Stool character score	6.19 ± 0.74	6.05 ± 0.58	0.427

Data are presented as mean ± SD or number.

*H. pylori*:  *Helicobacter pylori*; BMI: body mass index.

**Table 4:** Main study outcomes in the *H. pylori*-eradicated group and the control group in patients of long-term follow-up.

	*H. pylori*-eradicated group	Control group	*p* value
Complete response	8/42	3/22	
Partial response	17/42	8/22	
Responders^*∗*^	25/42	11/22	0.466
Abdominal pain score	1.10 ± 0.91	1.27 ± 0.83	0.446
Bloating score	0.61 ± 0.61	1.25 ± 0.71	0.047
Stools per day	3.07 ± 1.44	3.23 ± 1.41	0.680
Stool character score	4.76 ± 1.32	4.91 ± 1.19	0.663

Data are presented as mean ± SD or number.

^*∗*^Responders included patients who showed complete or partial response.

*H. pylori*:  *Helicobacter pylori*.
